# Telomeric repeat-binding factor 2: a marker for survival and anti-EGFR efficacy in oral carcinoma

**DOI:** 10.18632/oncotarget.10005

**Published:** 2016-06-14

**Authors:** Yordan Benhamou, Vincent Picco, Hélène Raybaud, Anne Sudaka, Emmanuel Chamorey, Sanja Brolih, Martino Monteverde, Marco Merlano, Cristiana Lo Nigro, Damien Ambrosetti, Gilles Pagès

**Affiliations:** ^1^ CNRS UMR 7284/INSERM U1081, Institute for Research on Cancer and Aging of Nice, University of Nice Sophia Antipolis, Nice, France; ^2^ Biomedical Department, Centre Scientifique de Monaco, Principality of Monaco; ^3^ Odontology Department, Nice University Hospital, University of Nice Sophia Antipolis, Nice, France; ^4^ Central Laboratory of Pathology, University of Nice Sophia Antipolis, Nice, France; ^5^ Department of Pathology, Research and Statistics, Centre Antoine Lacassagne, Nice, France; ^6^ Cancer Genetics and Translational Oncology Laboratory, S. Croce & Carle Teaching Hospital, Cuneo, Italy; ^7^ Medical Oncology, Oncology Department, S. Croce & Carle Teaching Hospital, Cuneo, Italy

**Keywords:** prognostic factor, predictive factor, oral cancer, tumor microenvironment, targeted therapies

## Abstract

Oral Squamous Cell Carcinoma (OSCC) is the most common oral cancer worldwide. Treatments including surgery, radio- and chemo-therapies mostly result in debilitating side effects. Thus, a more accurate evaluation of patients at risk of recurrence after radio/chemo treatment is important for preserving their quality of life. We assessed whether the Telomeric Repeat-binding Factor 2 (TERF2) influences tumor aggressiveness and treatment response. TERF2 is over-expressed in many cancers but its correlation to patient outcome remains controversial in OSCC. Our retrospective study on sixty-two patients showed that TERF2 overexpression has a negative impact on survival time. TERF2-dependent survival time was independent of tumor size in a multivariate analysis. In vitro, TERF2 knockdown by RNA interference had no effect on cell proliferation, migration, senescence and apoptosis. Instead, TERF2 knockdown increased the expression of cytokines implicated in inflammation and angiogenesis, except for vascular endothelial growth factor. TERF2 knockdown resulted in a decrease vascularization and growth of xenograft tumors. Finally, response to erlotinib/Tarceva and cetuximab/Erbitux treatment was increased in TRF2 knocked-down cells. Hence, TERF2 may represent an independent marker of survival for OSCC and a predictive marker for cetuximab/Erbitux and erlotinib/Tarceva efficacy.

## INTRODUCTION

Head and neck cancer is the fifth most common cancer in France and 90% of them are Oral Squamous Cell Carcinomas (OSCC). Despite treatment (invasive surgery, radiotherapy and chemotherapy) the overall survival ranges from 12 to 50% at 5 years depending on the localization in the mouth [1]. Eighty percent of OSCCs are associated with over-expression and activation of the Epidermal Growth Factor Receptor (EGFR), Mitogen-Activated Protein Kinase (MAPK) and PI3 Kinase/AKT signaling pathways [2]. Telomeric Repeat Factor 2 (TERF2) is a component of the shelterin complex, which interacts with distal ends of chromosomes to protect them from being recognized as DNA double strand breaks by DNA damage repair systems [3]. TERF2 represents an essential link between telomeric DNA and other components of the shelterin complex. In normal cells, TERF2 loss of function leads to activation of DNA repair systems specifically at telomeric loci, leading to cell cycle arrest, senescence or cell death [4–8]. In contrast, over-expression of TERF2 in the skin is associated with increased tumorigenesis [9]. Over-expression of TERF2 is observed in a variety of human cancers, suggesting that TERF2 plays a key role in tumor initiation and development [10–17]. However, the link between TERF2 expression in tumor tissues, overall survival and treatment response remains unclear. In addition, the potential of TERF2 as a prognostic marker or a predictive marker of sensitivity/resistance to targeted therapies has not been studied.

The end point of our study was to understand the relationship between the levels of TERF2 expression in OSCC and the i) aggressiveness of tumors and ii) their response to targeted therapies (e.g., cetuximab/Erbitux and erlotinib/Tarceva). Our results illustrate that overexpression of TERF2 in OSCC is a predictor of poor prognosis independent of tumor size. Additionally, levels of TERF2 protein expression are indicative of sensitivity to targeted therapies, *in vitro*. In the evolving era of personalized medicine, our results suggest that TERF2 expression analysis may help refine a cohort of patients likely to respond to commonly used EGFR-targeted therapies.

## RESULTS

### TERF2 is a prognostic marker of survival for OSCC

**Figure 1:**
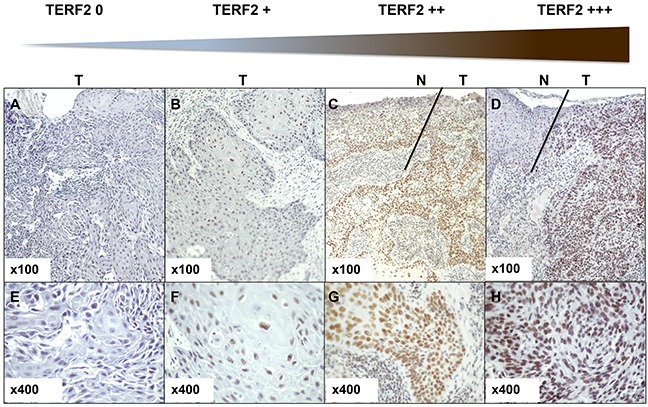
Determination of the TERF2 expression score. Immunohistochemical staining for TERF2 shows different expression levels in tumor cells from TERF2 0 to TERF2 +++. **A–C.** Panels indicate 100x magnification and E-H 400x magnification. N indicates normal tissue and T tumor tissue. Variation in the immunohistochemical stain was quantified by multiple lectures by three pathologists (DA, HR and AS). The different levels of staining and the number of cells stained in the tumor sections were taken into account to define scores from 0 to +++ (0 absence of nuclear staining; +1 weak nuclear staining; +2 At least 30% of tumor cells with a moderate nuclear staining; +3 At least 30% of tumor cells with a strong nuclear staining

**Figure 2:**
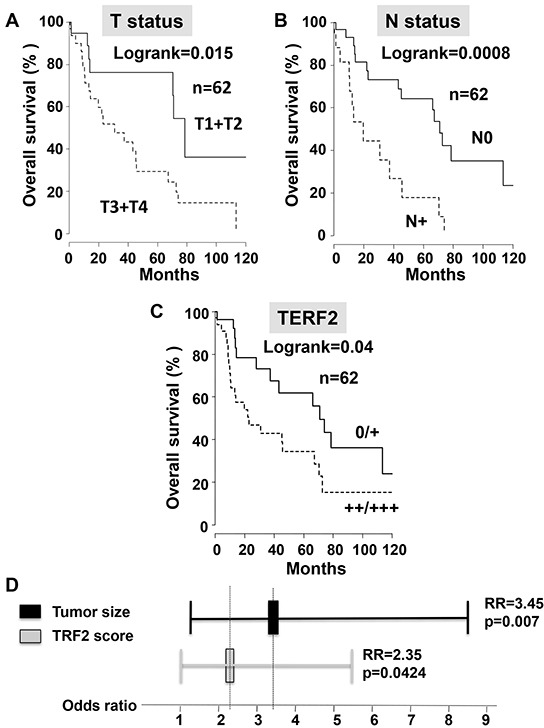
TERF2 is a marker of poor prognosis that is independent of the tumor size. **A–C.** Univariate survival analysis investigating the impact of the tumor size (T status), the nodal status (N status) or TERF2 expression on overall survival of patients with OSCC. **D.** Odds ratio for tumor size and TERF2 expression

We established a score of expression for TERF2 inspired from HER2 evaluation in breast cancers (proportion of labeled cells and labeling intensity) to standardize TERF2 detection in OSCC (Figure 1). The tumor size (T) and nodal status (N) significantly correlated to overall survival (*P* = 0.015 and 0.0008, respectively) (Figure 2A and 2B). 34 patients were scored TERF2 positive and 28 patients TERF2 negative. A significant relationship between TERF2 nuclear expression in OSCC tissue sections and survival was determined by an univariate analysis (Figure 2C) (median survival time 71 months for 0-1+ patients versus 24 months for 2+-3+ patients *P* = 0.0418). A multivariate analysis showed that the TERF2 score (OR = 2.35 [1.01 – 5.45] 95% CI, *P* = 0.0424) was independent of tumor size (OR = 3.45 [1.387 – 8.628] 95% CI, *P* = 0.007) (Figure 2D) introducing a new biological prognostic marker of survival for OSCC. In order to validate this result on independent cohorts, we performed *in silico* analysis using open access databases. Notably, TERF2 mRNA overexpression is inversely related to overall survival in head and neck squamous cell carcinoma, which strongly supports our results on an independent cohort of patients. Moreover, TERF2 mRNA expression is inversely related to survival in breast carcinoma (*P* = 0.045), colon carcinoma (Overall survival; *P* = 0.008; Disease free survival; P < 0.001) and prostate adenocarcinoma (Overall survival; P = 0.002). Alternately, TERF1 (an homologue of TERF2 present in the shelterin complex) and TERF2 expression levels were directly related to survival in lung adenocarcinoma (TERF2, disease free survival; *P* = 0.0097) and lung squamous cell carcinoma (TERF1, overall survival; *P* = 0.0065) (Table 1).

**Table 1:** *In silico* analysis of the effect of TERF1 and TERF2 expression levels on overall survival and disease free survival (http://www.cbioportal.org)

Cancer type	TERF2				TERF1			
	n	Overall survival	n	Disease free survival	n	Overall survival	n	Disease free survival
Breast invasive carcinoma	1092	High expression *Sup 1.5* p=0.045		NS		NS		NS
Colon carcinoma	374	High expression *Sup 3* p=0.008	330	High expression *Sup 3* p<0.001		NS		NS
Clear cell renal cell carcinoma		NS	435	High expression *Sup 1.1* p=0.044	532	High expression p=0.016	434	High expression p=0.036
Papillary renal cell carcinoma		NS	267	High expression *Sup 2* p=0.012	288	High expression p<0.001	267	High expression p<0.001
Esophageal carcinoma		NS		NS	193	High expression, *Sup 1.1* p=0.025		NS
Head and neck squamous cell carcinoma	517	High expression *Sup 1.1* p=0.0474		NS		NS		NS
Lung adenocarcinoma		NS		Low expression *Inf 1.5* p=0.0097		NS		NS
Lung squamous cell carcinoma		NS		NS		Low expression *Inf 3* p=0.0065		
Prostate adenocarcinoma	496	High expression *Sup 1.1* p=0.002		NS		NS	490	High expression *Sup 3* p=0.01
Uterine carcinoma		NS		NS		NS	162	High expression *Sup 2.5* p=0.009
Uveal melanoma		NS		NS	68	High expression *Sup 2* p=0.022	62	High expression *Sup 2* p=0.0015

The prognostic value for overall survival and disease free survival of RNA expression levels of TERF2 and its homologue TERF1 were obtained from publicly available databases. Cancers in which overexpression of the genes is detrimental are noted as “High expression” and cancers in which a lower expression is detrimental are noted “low expression” (grey background). Head and neck squamous cell carcinoma are presented on a black background. The p-values and threshold values (Sup or Inf) are indicated together with the number of patients (n). NS: non-significant.

### Effect of modulation of the TERF2 expression/activity on OSCC cell lines

**Figure 3:**
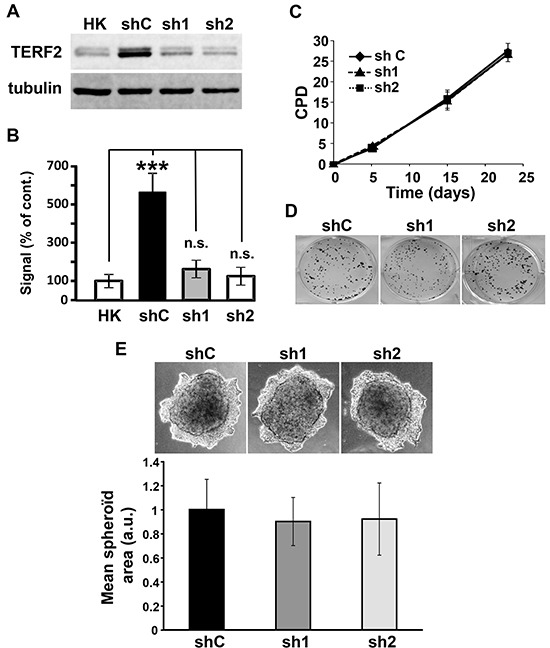
TERF2 down-regulation does not alter proliferation and invasion of CAL33 cells. **A.** Expression of TERF2 was tested in human keratinocytes (HK), CAL33 cells expressing scramble (shC) or two independent shRNA directed against TRF2 (sh1, sh2). Tubulin is shown as a loading control. **B.** Quantification of the blot shown in A: ***P < 0.001. **C.** Cumulative population doublings (CPD) of shC, sh1 and sh2 CAL33 cells. **D.** Clonal growth of shC, sh1 and sh2 CAL33 cells. **E.** Invasive properties of shC, sh1, sh2 CAL33 cells in matrigel. Quantification of the spheroid area of three independent experiments is shown

We next characterized the role of TERF2 in the proliferation abilities of OSCC cell lines. CAL33 cells showed a significantly higher TERF2 expression compared to primary human keratinocytes used as control normal cells (Figure 3A and 3B). Two independent shRNA sequences were used to knock-down TERF2 expression in CAL33 cells (Figure 3A and 3B). CAL33 cells over-expressing a wild-type or a dominant negative form of TERF2 were also generated (Supplementary Figure S1A). Modulation of TERF2 expression or activity did not influence the proliferative and invasive capacities or the DNA damage level of CAL33 cells (Figure 3C-3E, Supplementary Figure S1B and Supplementary Figure S2). Equivalent results were obtained for CAL27 cells (Supplementary Figure S1C-S1F).

### TERF2 down-regulation modified the secretome of the tumor cells

The above results suggest that the adverse effects linked to high expression of TERF2 on patients’ survival may not depend on the intrinsic properties of the tumor cells. Instead, TERF2 may influence the expression of factors that act on cells of the tumor microenvironment.

Therefore, we measured the cytokine levels in the supernatants of CAL33 cells with or without TERF2 knock-down (Supplementary Figure S3). TERF2 knock-down in CAL33 resulted in the induction of CXCL1, CXCL8, CXCL9, CXCL10, interleukin 6 (IL6), PDGF-BB and RANTES and a decrease in VEGF expression (Table 2). CXCL 8, 9 and 10 were similarly modified in the CAL27 (Table 1). CXCL1 and CXCL7, modified respectively in CAL33 and CAL27, may be interchangeable because they share similar activities and stimulate the same G protein coupled receptors [23]. These results suggest that TERF2 acts as a gene expression regulator.

**Table 2:** Effect of TRF2 silencing on expression levels of angiogenic and inflammatory genes in CAL33 cells, CAL27 cells and CAL33 tumor xenografts

	CAL33				CAL27		
	Genes	shC	sh1	sh2	shC	sh1	sh2
CELLS	36B4	100	100	100	100	100	100
	m-RPLP0	100	100	100	100	100	100
	GADPH	100	100	100	100	100	100
	TERF2	100	70(*)	40(***)	100	62(*)	32(***)
	CXCL1	100	326(***)	307(***)	100	129	80
	CXCL7	100	110	131	100	199(*)	379(***)
	CXCL8	100	357(***)	197(***)	100	212(*)	142(**)
	CXCL9	100	235(**)	334(***)	100	293(*)	355(**)
	CXCL10	100	162(*)	479(***)	100	271(**)	267(**)
	IL6	100	400(***)	697(***)	100	289(***)	100
	PDGF-BB	100	122(*)	150(***)	100	130	93
	RANTES	100	270(***)	310(***)	100	97	86
	VEGF	100	85	68(***)	100	109	82
TUMORS	36B4	100	100	100			
	TERF2	100	91 (*)	51 (***)			
	CXCL1	100	276 (***)	302 (**)			
	CXCL7	100	124	125			
	CXCL8	100	96	119			
	CXCL10	100	179 (*)	1261 (*)			
	IL6	100	139	193			
	RANTES	100	518 (***)	1325 (**)			
	VEGF	100	48 (*)	57 (**)			

The percentage expression of the different genes evaluated by qPCR is shown. For the measured genes, the reference values (100) correspond to the content of a given gene in shC cells. The statistically significant differences are shown (ANOVA test). * p < 0.05: ** p < 0.01: *** p < 0.001.

### TERF2 knock-down decreased the growth of OSCC xenografts in mice

**Figure 4:**
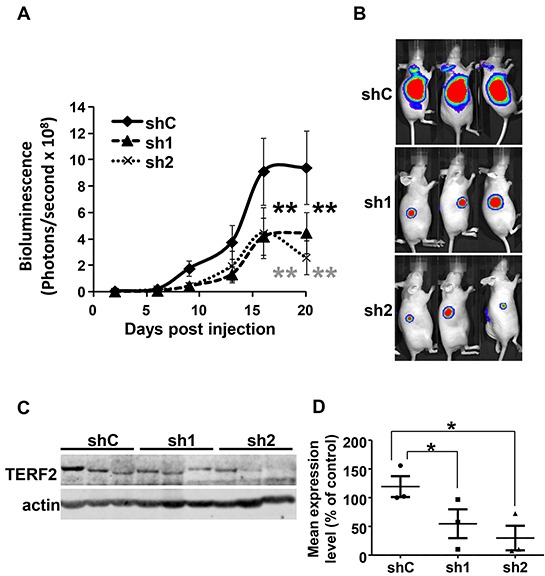
Figure 4: TERF2 down-regulation decreased tumor growth. **A.** 10^6^ CAL33-Luc cells expressing shC, sh1 or sh2 were subcutaneously injected into nude mice (n = 10 per group). Bioluminescence was measured weekly as described previously [49]. Results are presented as the mean ± SD. Statistical differences between the size of tumors of shC, sh1 and sh2 mice are presented: ** *P* < 0.01. **B.** Representative images of tumor-bearing mice. **C.** TERF2 was analysed by immunoblotting in three independent tumors for each condition (shC, sh1 and sh2). **D.** Quantification of the signals shown in C (* P < 0.05)

TERF2-dependent tumor aggressiveness was tested by generating tumors in nude mice with CAL33 expressing the luciferase gene (CAL33-Luc cells) and control or two independent TERF2-directed shRNA sequences. Tumors with TERF2 knock-down were smaller (Figure 4A and 4B). The smallest tumors were associated with the highest TERF2 knock-down (sh2 group) (Figure 4A, 4C and 4D). TERF2 knock-down was confirmed by quantification of immunoblots (Figure 4C and 4D).

### TERF2 knock-down prevented blood vessel organization and favored fibrosis, inflammation and tumor necrosis

**Figure 5:**
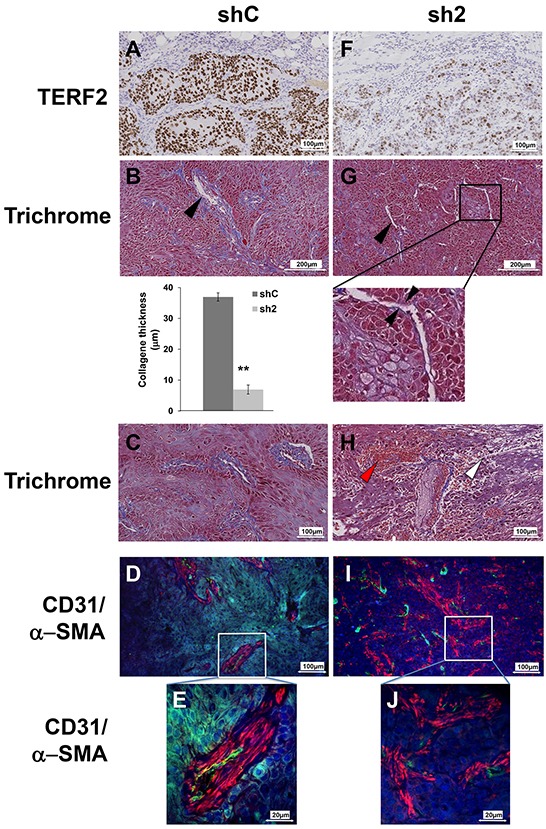
TERF2 down-regulation destabilized tumor vessels and induced fibrosis. **A, F.** Immunohistochemical staining for TERF2. **B, G.** General histological aspect of shC or sh2 tumors stained with trichrome. The black arrowhead shows collagen surrounding the vessels. Mean collagen thickness around vessels was measured in shC and sh2 tumor sections. **C, H.** Trichrome staining also shows immune cell extravasation (white arrowhead) and blood cells extravasation (red arrowhead). **D, I.** Tumor vasculature was detected with CD31 (endothelial cells, green) and α-SMA immuno-staining (pericytes, red) and nuclei were labeled with DAPI (blue). Higher magnification showing pericyte coverage of blood vessels in shC tumors E. and diffuse pericytes in sh2 tumors J

Sections from tumors generated with control or knock-down cells for TERF2 were analyzed to understand the mechanism associated with reduced tumor growth (Figure 5). Compared to control tumors (Figure 5A), tumors in which TERF2 was silenced (Figure 5F) were characterized by necrotic zones (7.5% versus 26%, *P* = 0.018, Supplementary Figure S4) and a thinner layer of collagen around vessels (37 μm versus 7 μm, *P* = 0.001, Figure 5B, 5G). Moreover, inflammatory and red blood cell extravasation was observed around vessels in TERF2 knocked-down tumors, suggesting the presence of acute inflammation and a disorganized vascular network (Figure 5C and 5H). Tumor sections were monitored for vascularization by labeling for CD31 (endothelial cells) and (pericytes) α-SMA. Control tumors were characterized by a high blood vessel density with coverage of endothelial cells with pericytes (Figure 5D and 5E). The vascular network of shTERF2 tumors was disorganized with dispersed endothelial cells and pericytes, a characteristic of fibrotic zones (Figure 5I and 5J). This disorganization is consistent with VEGF down-regulation and CXCL10 induction observed in cultured cells and tumor xenografts (Table 2).

### CAL33 cells knocked-down for TERF2 are more sensitive to erlotinib/Tarceva and cetuximab/Erbitux

**Figure 6:**
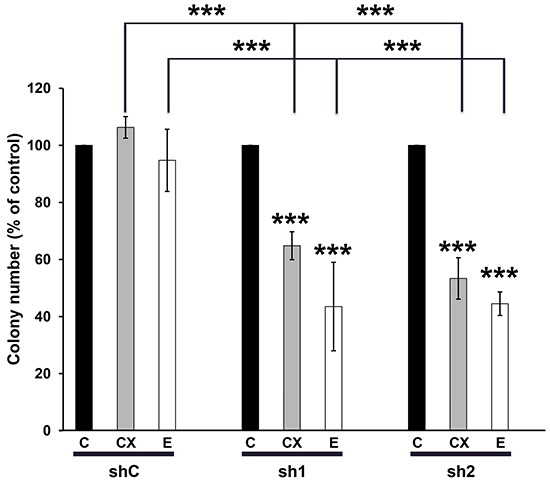
TERF2 down-regulation sensitized CAL33 to erlotinib/Tarceva and cetuximab/Erbitux. Clonal growth of CAL33-shC, sh1 and sh2 cells in the absence or presence of 6 nmol/L cetuximab (CX) or of 0.1 mol/L erlotinib (E). The number of colonies in the absence of drugs for each cell line was considered as the reference value (100%). ***P < 0.001

OSCC are characterized by over-expression of the Epidermal Growth Factor Receptor (EGFR) and activation of downstream signaling pathways. Hence, cetuximab/Erbitux is prescribed to patients with loco-regionally advanced cancers and to elderly patients with kidney insufficiency. Cetuximab/Erbitux is also given to metastatic patients at progression on radio/chemotherapy (5Fluorouracyl/platinum salts) or as a mono-therapeutic agents for patients in poor general conditions or for elderly patients [24–25]. We hypothesized that TERF2 expression may influence the response to inhibitors of EGFR erlotinib/Tarceva and cetuximab/Erbitux. Erlotinib/Tarceva efficiently inhibited the activity of EGFR and downstream signaling pathways (ERK and AKT) of CAL33 cells (Supplementary Figure S5). The knock-down of TERF2 had no influence on the efficacy of radiotherapy or 5-Fluorouracil treatment (Supplementary Figure S6A and S6B). However, it increased the efficacy of erlotinib/Tarceva (0.1 mol/L) and cetuximab/Erbitux (6 nmol/L) at doses largely inferior to the IC50 for these two drugs (respectively 5.4 mol/L [26] and 30 nmol/L [27]) (Figure 6). MTT assays confirmed that erlotinib/Tarceva inhibited CAL33 cell proliferation when TERF2 was knocked-down (Supplementary Figure S6C). The knock-down of TERF2 also increased the efficacy of cetuximab/Erbitux in CAL27 cells (Supplementary Figure S6D). These results suggest that TERF2 expression may constitute a relevant predictive marker of anti-EGFR treatments in OSCC.

## DISCUSSION

We created a reading score for immunohistochemistry-based detection of TERF2 expression levels using a commercially available monoclonal antibody. Importantly, this prognostic score is readily usable by pathologists and the staining can be performed on diagnostic biopsies. Its routine detection could be informative as a new biological parameter to help take decisions concerning treatment. OSCC patients undergo surgery, then radiotherapy associated or not with chemotherapy. These treatments are known to induce major debilitating side effects such as mucitis. It is therefore important to identify patients with a better prognosis to administer them a less aggressive treatment [28–29].

Two previous studies assessed TERF2 expression in OSCC and reported discrepant results [30–31]. Consistent with one of these studies, we observed significant TERF2 over-expression in OSCC [30–31]. The Chinese, European and Indian ethnicities of the patients of the studies may explain the different TERF2 expression levels. The differentiation grade, not stated by Chuang and colleagues, could also explain the differences as TERF2 expression is greater in the basal layer of the epithelium and decreases in cells of the superficial layers [10, 32–33].

Low and high levels of TERF2 did not modify proliferation, migration and invasion. Instead, decreased TERF2 expression had a broad effect on the cellular secretome [19]. Modification of cytokine expression suggested that a senescence-associated-secretory phenotype may be associated with TERF2 inhibition [34]. However, we never observed obvious signs of senescence (increase in cell size and spreading, increase in the size of nuclei or polynucleation), DNA damage (nuclear 53BP1 foci) or apoptosis (poly ADP ribose polymerase cleavage). The genes induced by TERF2 down-regulation are involved in the angiogenic balance and inflammatory processes. The decrease in VEGF expression and the increase in CXCL9 and CXCL10 expression may account for the impaired vascularization of experimental tumors [23]. The implication of TERF2 in angiogenic processes has been described recently through direct regulation of the platelet-derived growth factor receptor β in endothelial cells, but not directly in the tumor cells [35]. TERF2 high expression levels correlate with lower expression of a variety of pro-inflammatory and immune system activating cytokines (IL6, IL8/CXCL8, RANTES/CCL5 and GRO/CXCL1). Their high expression is commonly associated with poor prognosis in cancers [36–39]. However, CCL5 was described as a beneficial factor for cancer immunotherapy [40]. It participates in activation of natural killer (NK) cells. Notably, NK cell activation has previously been associated to a decrease in TERF2 tumor expression [41–42]. IL6 can promote B-cell differentiation, which is thought to prevent tumor growth [43]. Finally, CXCL1 and CXCL8 are major chemo-attractants for leukocytes and play a key role in immune depletion of tumors [44–45]. Therefore, high expression of TERF2 in tumors may repress of a panel of cytokines that enhance an anti-tumor immune response.

PDGF-BB, a growth factor normally released by activated platelets, is down-regulated in cells and tumors expressing high levels of TERF2. This factor is pro-tumoral in epithelial cancers [46]. However, PDGF-BB over-expression inhibits the growth of angiogenesis-dependent tumors [47]. We also observed an increase in the pericyte content that did not co-localize with the blood vessels in tumors expressing low levels of TERF2. Hence, an increased pericyte number could account for the lower growth of the tumors with TERF2 knock-down.

Our experiments suggested that TERF2 may represent a relevant predictive marker for treatment with cetuximab/Erbitux and erlotinib/Tarceva. We tried to confirm our *in vitro* results on a cohort of 16 OSCC patients who had received cetuximab/Erbitux. Unfortunately, we could not demonstrate a predictive role of TERF2 expression. The specific characteristic of these patients that have a reduced survival time compared to the general population of OSCC patients may have biased the predictive role of TERF2. The relevance of the TERF2 expression level as a predictive marker of anti-EGFR therapies may therefore have to be evaluated in a prospective study. Together, our pioneering experiments may constitute a valuable tool in determining the patients at risk of recurrence and stratifying patients who can benefit from anti-EGFR targeted therapies.

## MATERIALS AND METHODS

### Patients and tissue samples

Sixty-two diagnostic biopsies of OSCC were obtained at the Centre Antoine Lacassagne (CAL) and Hospital St Roch between 1996 and 2011. OSCC was confirmed by histology. Patients gave their consent for the study, which was approved by our institutional review board. Tumor sections were evaluated by immunohistochemistry. The TNM classification, diagnosis, date of diagnosis, treatment and last known status of the patient were obtained by searching through the clinicom ® database. Survival was calculated from the date of diagnosis. Key patient characteristics are summarized in Table 3. The mean age of patients was 60.5 years. Most patients were men (71%) with a sex ratio of 2.45. Most of the tumors were invasive (44%) (T4 stage) and measured more than 4cm. Sixty-three percent of the patients did not present lymphatic node invasion. Most of the patients were treated by surgery (67%) and radiotherapy (67%) and chemotherapy wasused in only 14 patients (25%). The median survival time was 45.3 months with a 1 year survival rate of 78.5%.

**Table 3:** Patients clinical characteristics

Variable	Number	Frequency (%)
**Sex**		
Male	44	71
Female	18	29
**T stage**		
Unknown	10	16
1	12	19
2	8	13
3	9	15
4	23	37
**N stage**		
Unknown	13	21
0	31	50
1	2	3
1c	1	2
2	3	5
2b	7	11
2c	3	5
3	2	3
M stage		
Unknown	21	34
0	41	66
**Differentiation**		
Unknown	30	48
High Grade Dysplasia	1	2
1	5	8
2	5	8
3	21	34
**Keratinization**		
Unknown	32	52
No	10	16
Yes	20	3(Continued )2
**Inflammation**		
Unknown	39	63
0	11	18
1	4	6
2	8	13
**Surgery**		
Unknown	4	6
Surgery	39	63
No surgery	19	31
**Radiotherapy**		
Unknown	5	8
Radiotherapy	38	61
No radiotherapy	19	31
Chemotherapy		
Unknown	5	8
Chemotherapy	14	23
No chemotheapy	43	69

### Cell lines

Two human head and neck cancer cell lines, CAL33 and CAL27 [18] were provided through a Material Transfer Agreement with the Oncopharmacology Laboratory, Centre Antoine Lacassagne [18]. Human primary keratinocytes were kindly provided by Dr Thierry MAGNALDO and Dr Maria GONCALVES-MAIA of our Institute. For knock-down experiments, the cells were infected using a lentiviral vector containing non-target control or two independent sequences of shRNA targeting the *terf2* gene (sh#1 5′-GCCAGAATATCATTAGCGTTT-3′ and sh#2 5′-GCGCATGACAATAAGCAGATT-3′) cloned in pLKO.1 puro, (gift from Bob Weinberg, Addgene plasmid #8453). For ectopic expression experiment, the cells were infected with an empty pWPIR vector containing an IRES-driven GFP gene or wild-type or dominant negative form of TERF2 [19]. The GFP-positive cells were sorted by cytometry. Luciferase expressing cell lines (CAL33-Luc) were generated by lentiviral infection with pLenti CMV V5-Luc (Addgene plasmid 21474).

### Proliferation assays

Population doublings were calculated as previously described [20]. The mean and standard deviation were calculated for three independent experiments. For clonogenicity assays, 2 × 10^3^ cells were seeded onto 60-mm dishes. Twenty-four hours after, medium was replaced with DMEM supplemented with 7.5% heat inactivated fetal calf serum (FCS) in the presence or absence of erlotinib/Tarceva (1 mol/L) or cetuximab/Erbitux (6 nmol/L). Cells were grown for 10 days. Cells were stained with Giemsa (Fluka). The plates were scanned and analyzed with ImageJ software (NIH, USA). The concentration of erlotinib/Tarceva that decreased cell growth was assessed using the 3-[4,5-dimethylthiazol- 2yl]-diphenyltetrazolium bromide (MTT) colorimetric assay (Sigma, Lyon, France) according to the manufacturer's instructions.

### Cell migration and invasion

4000 cells were seeded in 20μL hanging drops of DMEM supplemented with 7.5% FCS to obtain spheroids. After 7 days, they were transferred in DMEM-3% FCS supplemented with 1μg/mL matrigel (Corning Inc) and cultured for 15 days. Pictures were taken with an AMG Evos microscope 40x objective (Thermo Fisher Scientific Inc) and the spheroid areas were measured using ImageJ software (NIH, USA).

### Immunoblotting

The following antibodies were used: anti-phospho ERK1/2 (Sigma St Louis, MO), anti-phospho AKT, anti-AKT, anti-EGFR (Cell Signaling, Cambridge, UK,), anti-ERK1/2 (Santa Cruz Biotechnology, Santa Cruz, CA), anti-TERF2 (Novus bio, Cambridge, UK) and α-tubulin (Fischer scientific, Illkirch France).

### Tumor formation and size evaluation

10^6^ cells were injected subcutaneously into the flanks of 5-week-old nude (nu/nu) female mice (Janvier). Bioluminescence was quantified using the In Vivo Imaging System (Perkin Elmer) according to the manufacturer's instructions. Tumor volume (v = × l^2^ × 0.52) was determined with a caliper. A linear relationship exists between values for bioluminescence and the tumor volume. This study was carried out in strict accordance with the recommendations in the Guide for the Care and Use of Laboratory Animals. Our experiments were approved by the “Direction de l'Action Sanitaire et Sociale (DASS)” of the Principality of Monaco and the ethic committee of our Institute.

### Pathology techniques

Pathological analysis was performed on 3 μm tissue sections colored with Masson's trichrome (blue collagen) and scanned with a Leica Slide Path. The following parameters were analyzed using Leica Slide Path Gateway software: area of the section, area of necrosis, presence of white blood cell infiltrates, presence of red blood cell extravasation, thickness of collagen around vessels, number of vessels. Immunohistochemistry was carried out on 3 μm tissue sections of formalin-fixed, paraffin-embedded tissue blocks. Endogenous peroxidase inactivation for 30 minutes of deparaffinized sections was carried out following re-hydratation (Dako 48 link autostainer, Dako, Capinterie, CA, USA) and heat unmasking of antigens for 20 minutes at 97°C in a pH9 buffer solution (PT link Dako device). Incubation was carried out with monoclonal anti-TERF2 antibody diluted at 1:100 for 20 minutes at room temperature. Tissues sections were then treated with 3′,3′-diaminobenzidine chromogen and counterstained for the nucleus with Mayer's hematoxylin. The TERF2 reactivity on lymphocytes and/or basal epithelial cells was considered as an internal positive control. Nuclear expression of TERF2 was semi-quantitatively analyzed and verified independently by two pathologists (D. Ambrosetti and A. Sudaka) and two surgeons (H. Raybaud (also qualified as a pathologist) and Y. Benhamou). In cases of discrepancies, a fifth person was consulted. Tumors were classified as: 0 absence of nuclear staining in tumor cells (negative); +1 weak, barely perceptible nuclear staining (negative); +2 At least 30% of tumor epithelial cells have a moderate nuclear staining (positive); +3 At least 30% of tumor epithelial cells have a strong to intense nuclear staining (positive). When tissue sections contained two scores, the upper score was chosen if 30% or more stained nuclei were concerned.

### Immunofluorescence

Analysis of tumor sections was performed as previously described (26). Sections were incubated with a rat monoclonal anti-mouse CD31 (clone MEC 13.3; BD Pharmingen) and a monoclonal anti--smooth muscle actin (1:1000; A2547Sigma, France). Analysis of cell lines was carried out using a standard protocol with anti-53BP1 (1/500, MB100-305 Novus Biological).

### Analysis of cbioportal databases

Overall survival was calculated from patient subgroups with head and neck squamous cell carcinoma (TCGA provisional) with mRNA levels of TERF2 that were 1.1 fold greater than the median value (mRNA Expression z-Scores RNA Seq V2 RSEM)). Head and neck tumor samples with mRNA data were selected in cbioportal (522 samples out of 530; 517 samples analyzed for survival). A specific threshold (lower (Inf) or greater (Sup) than the median was determined for each cancer and TERF1 and TERF2 to have the best p value. For each tumor type, samples with available mRNA data were selected.

### Statistical analysis

The end point for all analyses on patients was overall survival (OS). For live patients, the time from primary diagnosis to the last documented follow-up was used. The OS rates were calculated according to the Kaplan Meier method. The hazard ratio (HR) between different groups defined by the TERF2 score and confidence intervals was determined by the cox regression model. The categorization of the immunohistochemistry factors in subgroups was predefined independently. For univariate and multivariate analyses, the 0 and 1+ and the 2+ and 3+ tumors were combined in independent groups representing “TERF2 negative” and “TERF2 positive”. Statistical analyses were two sided and performed using R-3.0.2 for Windows.

## SUPPLEMENTARY FIGURES


