# Supplementary material for: Telomeric repeat-binding factor 2: a marker for survival and anti-EGFR efficacy in oral carcinoma
Source: Oncotarget. 2016 Jun 14;7(28):44236–51. doi: 10.18632/oncotarget.10005 (PMC5190092; doi:10.18632/oncotarget.10005)
Supplement: Supplementary file 1 [file oncotarget-07-44236-s001.pdf]

Telomeric repeat-binding factor 2: a marker for survival and anti-EGFR efficacy in oral carcinoma

SUPPLEMENTARY FIGURES

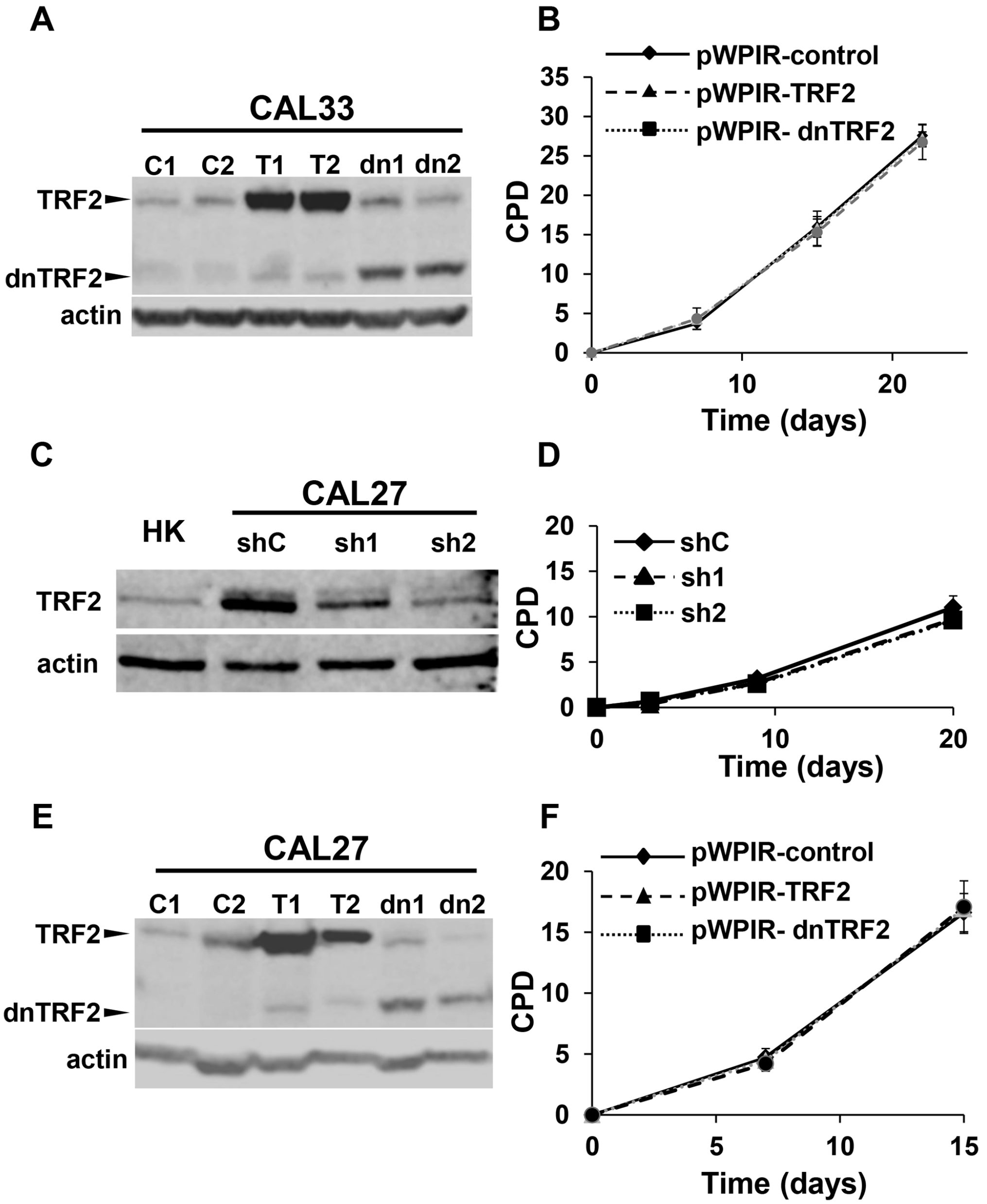

Supplementary Figure S1: Expressions of wild-type or a dominant negative form of TERF2 and TERF2 down-regulation do not impair CAL33 and CAL27 proliferation. A. CAL33 cells stably transfected with a control expression vector (pWPIR-control; C1 and C2), a vector for TERF2 (pWPIR-TERF2; T1 and T2) or a dominant negative form of TERF2 (pWPIR-dnTERF2; dn1 and dn2) were tested for the presence of TERF2 by immunoblotting. Actin is shown as a loading control. **B.** Cumulative population doublings (CPD) of pWIR-control, pWIR-TRFR2 and pWIR-dnTERF2 CAL33 cells. **C.** Primary human keratinocytes (HK) and CAL27 cells expressing control (shC) or two independent shRNA sequences directed against TERF2 (sh1 and sh2) were tested for the presence of TERF2 by immunoblotting. Actin is shown as a loading control. **D.** Cumulative population doublings (CPD) of shC, sh1 and sh2 CAL27 cells. E. CAL27 cells stably transfected with a control expression vector (pWIR-control), a vector for TERF2 (pWIR-TERF2) or a dominant negative form of TERF2 (pWIR-dnTERF2) were tested for the presence of TERF2 by immunoblotting. Actin is shown as a loading control. **F.** Cumulative population doublings (CPD) of pWIR-control, pWIR-TRFR2 and pWIR-dnTERF2 CAL27 cells.

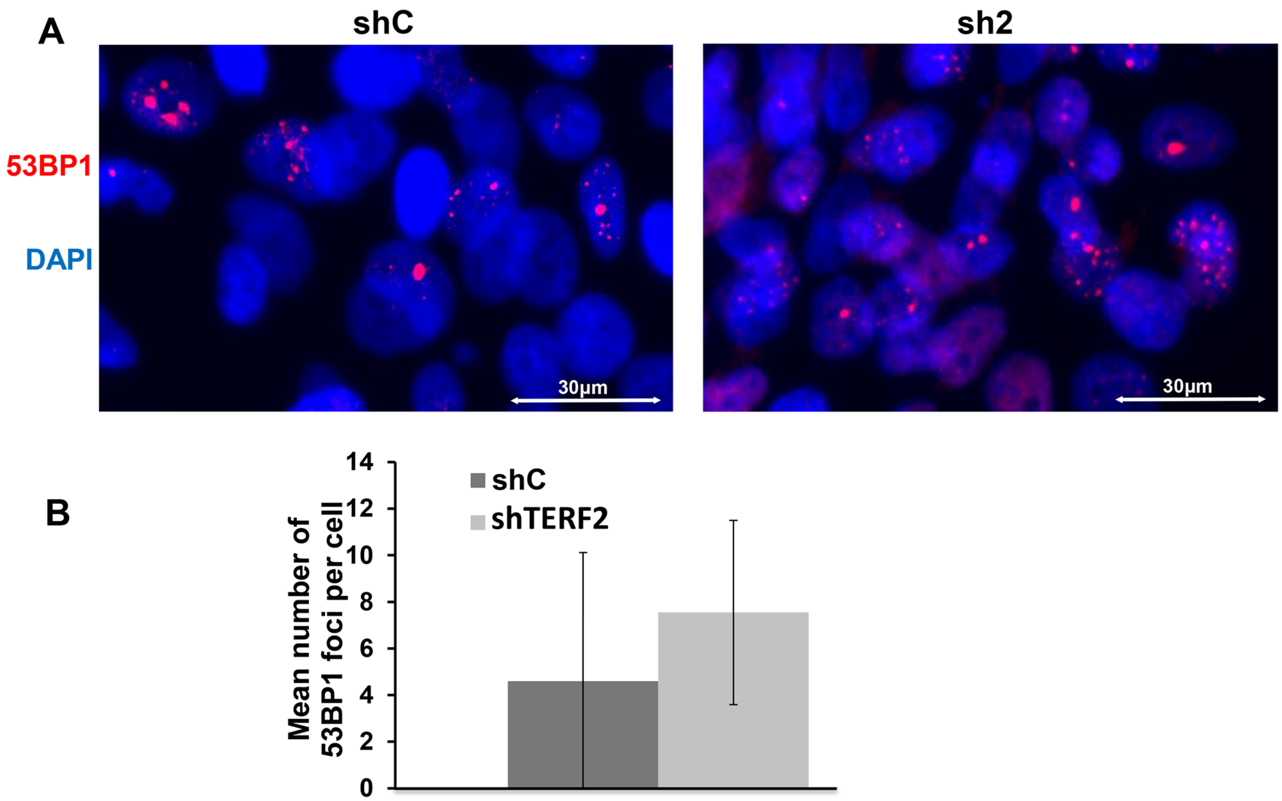

Supplementary Figure S2: Down-regulation of TERF2 has no incidence on DNA damage. A. CAL33 cells were analyzed for 53BP1 foci (red). The DNA was labeled with Hoechst dye (blue). **B.** Mean number of 53BP1 foci per cell. Results are presented as mean ± SD.

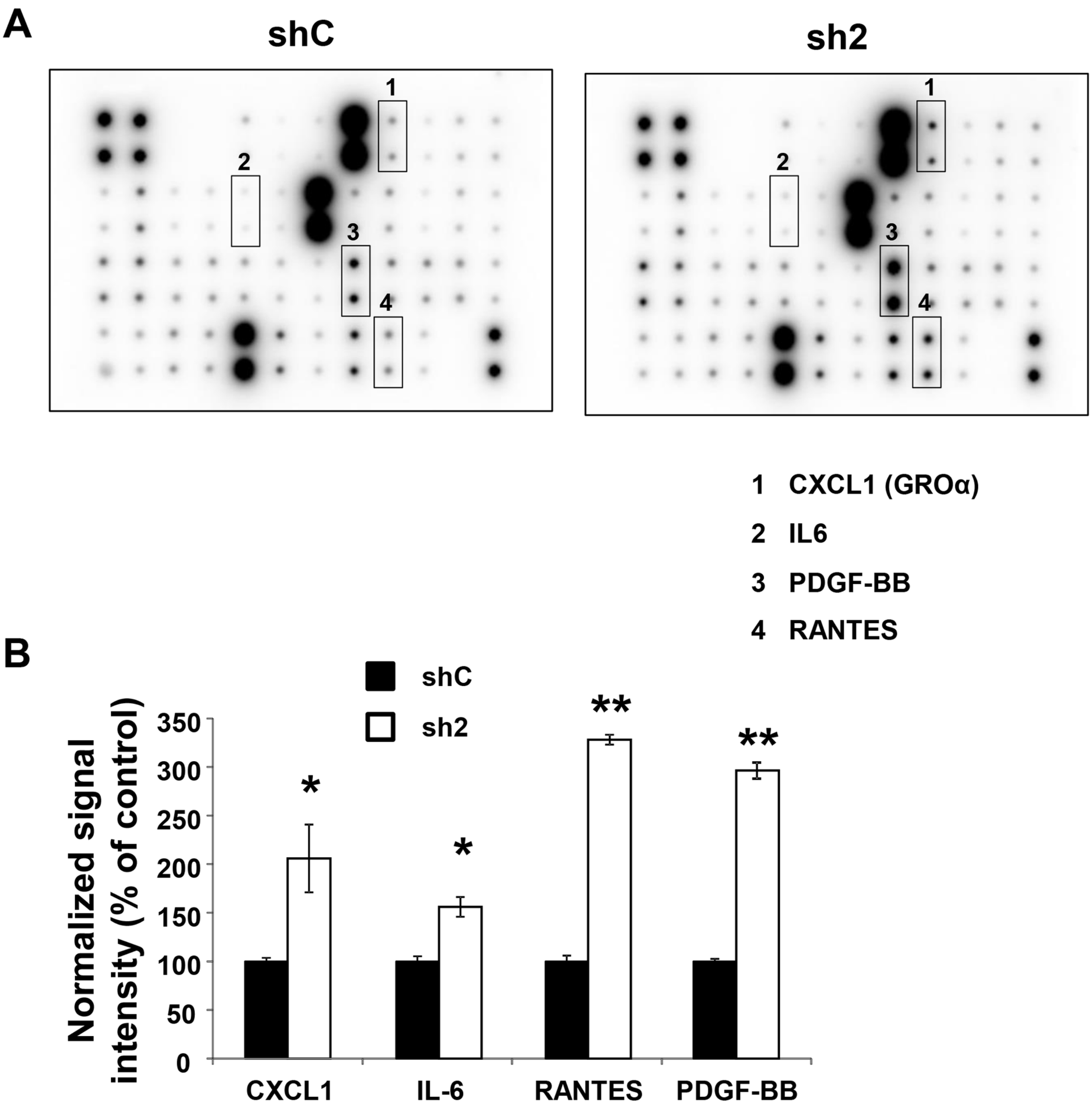

Supplementary Figure S3: Down-regulation of TERF2 modifies the secretome of CAL33. A. shC and sh2 CAL33 cell supernatants were tested for the presence of pro/anti-angiogenic, pro-inflammatory cytokines and growth factor using a sandwich ELISA macroarray. Cytokines with a differential signal between shC and sh2 supernatants were boxed (CXCL1, IL6, PDGF-BB and RANTES). **B.** Quantification of the signals shown in A. * $P< 0.05$; ** $P< 0.01$.

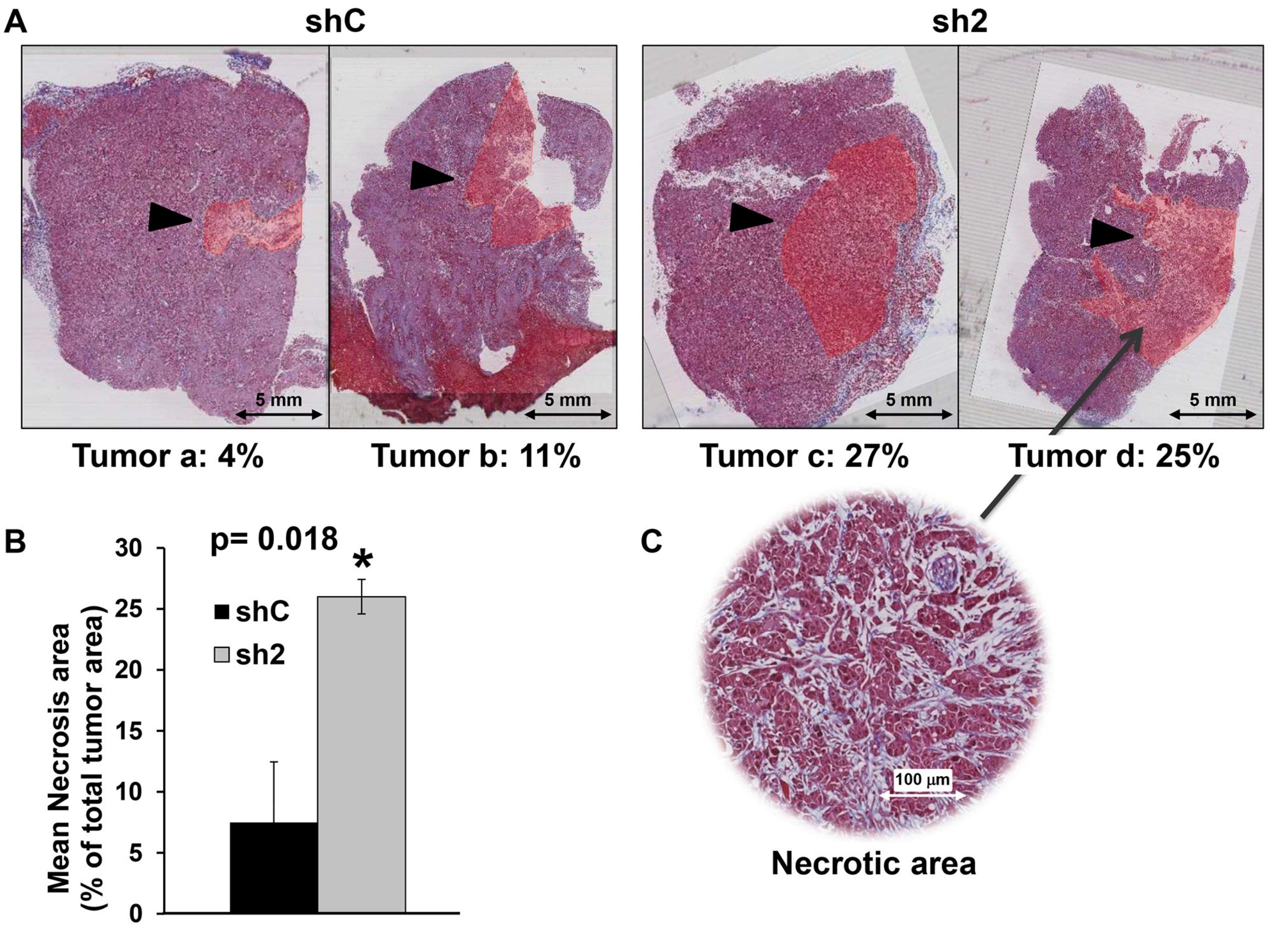

Supplementary Figure S4: Down-regulation of TERF2 induces tumor necrosis. A. Haematoxylin/eosin labelling of sections of tumors from shC and sh2 tumor reveals necrotic zones (arrowheads). The percentage of the necrotic zone relative to the total surface of the tumor section is shown. **B.** Mean necrotic area for shC and sh2 tumors (* P< 0.05). **C.** magnification of the indicated necrotic zone.

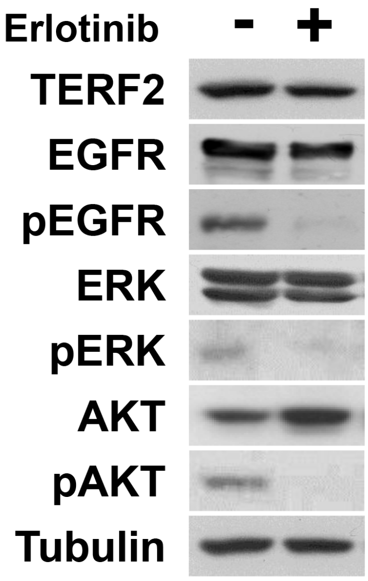

Supplementary Figure S5: Erlotinib inhibits EGFR in CAL33 cells. CAL33 cells were incubated in the absence (-) or presence (+) of erlotinib (10 mol/L). TERF2, EGFR, pEGFR, ERK, pERK, AKT, pAKT were analyzed by immunoblotting. Tubulin is shown as a loading control.

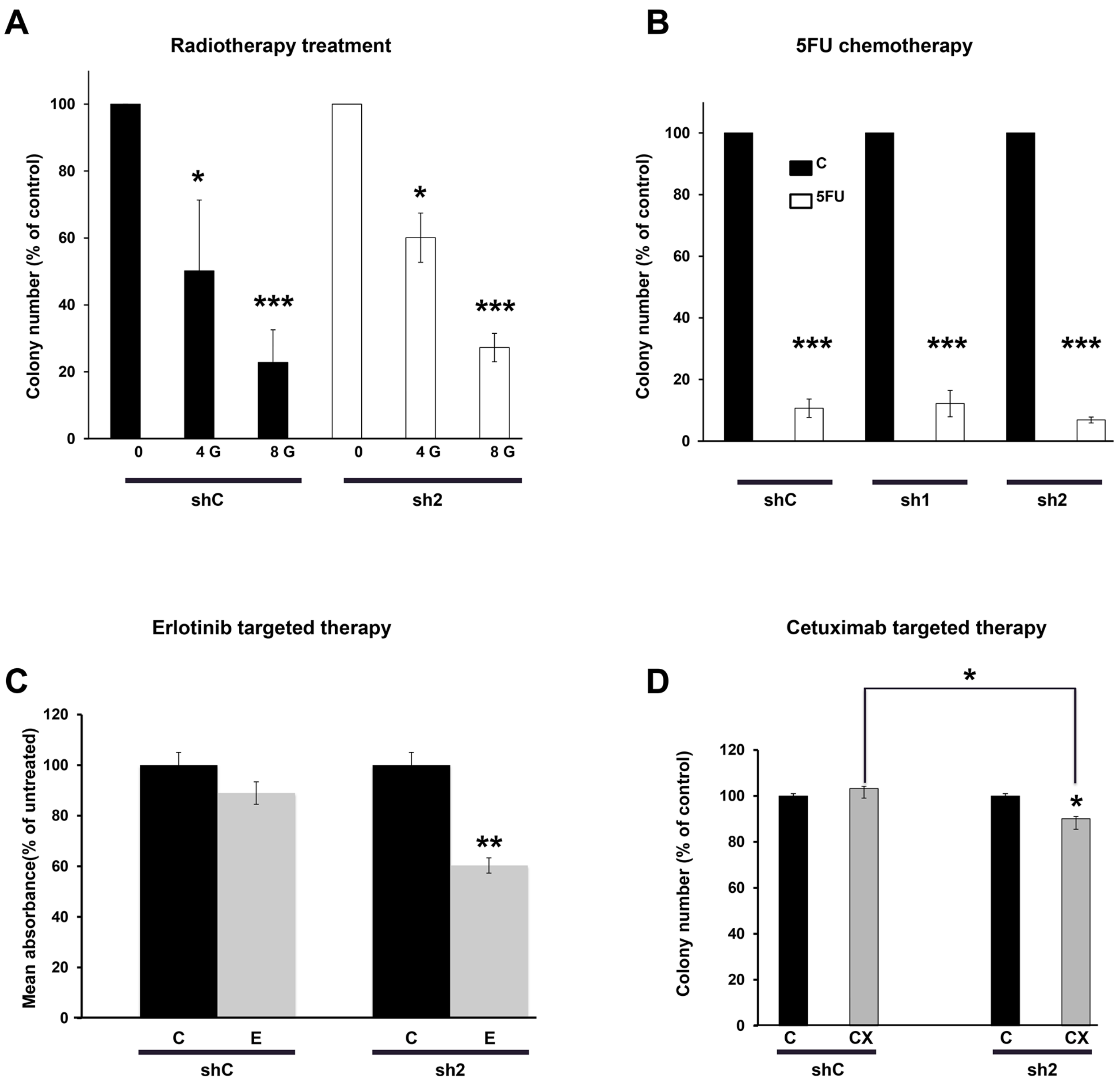

Supplementary Figure S6: Down-regulation of TERF2 does not modify the sensitivity to irradiation and 5FU but modifies sensitivity to erlotinib. A. Clonal growth of CAL33-shC and sh2 after the indicated doses of irradiation (4 and 8 grays). **B.** Clonal growth of CAL33-shC, sh1 and sh2 in the absence or presence of 0.01 mol/L 5FU. The number of colonies in the absence of drugs for each cell line was considered as the reference value (100%). (* P < 0.05; *** P < 0.001). **C.** MTT tests were performed on CAL33-shC and sh2 cells in the presence or absence of 0.1 mol/L erlotinib (E). The mean OD after four days of untreated cells is used as the reference value (100%). (** P < 0.01). **D.** Clonal growth of CAL27-shC and sh2 in the absence or presence of 1 µg/ml cetuximab (CX). The number of colonies in the absence of drugs for each cell line was considered as the reference value (100%). * P < 0.05.
